# Promoting physical activity through supervised vs motivational behavior change interventions in breast cancer survivors on aromatase inhibitors (PAC-WOMAN): protocol for a 3-arm pragmatic randomized controlled trial

**DOI:** 10.1186/s12885-023-11137-1

**Published:** 2023-07-05

**Authors:** Eliana V. Carraça, Bruno Rodrigues, Sofia Franco, Inês Nobre, Flávio Jerónimo, Vítor Ilharco, Fernanda Gabriel, Leonor Ribeiro, António L. Palmeira, Marlene N. Silva

**Affiliations:** 1 Centro de Investigação Em Educação Física, Desporto, Saúde e Exercício (CIDEFES), Universidade Lusófona, Campo Grande 376, Lisboa, 1749-024 Portugal; 2 CIAFEL, Faculdade de Desporto, Universidade do Porto, Porto, Portugal; 3 Programa Nacional Para a Promoção da Atividade Física, Direção-Geral Saúde, Portugal; 4 Faculdade de Motricidade Humana, Universidade de Lisboa, Cruz Quebrada, Lisboa, Portugal; 5 Centro Hospitalar Universitário Lisboa Norte – Hospital de Santa Maria, Lisboa, Portugal

**Keywords:** Survivorship, Aromatase inhibitors, Quality of life, Physical sctivity, Active lifestyle, Lasting behavior change, Self-determination theory

## Abstract

**Background:**

Aromatase inhibitors (AI) are frequently used to treat hormone-receptor-positive breast cancer, but they have multiple adverse effects (e.g., osteoporosis, arthralgia), resulting in premature therapy discontinuation/switch. Physical activity (PA) can attenuate these negative effects and improve quality of life (QoL). However, most cancer survivors fail to perform/sustain adequate PA levels, especially in the long-term. Theory-based interventions, using evidence-based behavior change techniques, aimed at promoting long-term behavior change in breast cancer survivors are effective, but remain scarce and fail to promote self-regulatory skills and better-quality motivations associated with sustained PA adoption. This paper describes the design of the PAC-WOMAN trial, which will test the long-term effectiveness and cost-effectiveness of two state of the art, group-based interventions encouraging sustained changes in PA, sedentary behavior, and QoL. Additional aims include examining the impact of both interventions on secondary outcomes (e.g., body composition, physical function), and key moderators/mediators of short and long-term changes in primary outcomes.

**Methods:**

A 3-arm pragmatic randomized controlled trial, involving a 4-month intervention and a 12-month follow-up, will be implemented, in a real exercise setting, to compare: 1) brief PA counseling/motivational intervention; 2) structured exercise program vs. waiting-list control group. Study recruitment goal is 122 hormone-receptor-positive breast cancer survivors (stage I-III), on AI therapy (post-primary treatment completion) ≥ 1 month, ECOG 0–1. Outcome measures will be obtained at baseline, 4 months (i.e., post-intervention), 10 and 16 months. Process evaluation, analyzing implementation determinants, will also be conducted.

**Discussion:**

PAC-WOMAN is expected to have a relevant impact on participants PA and QoL and provide insights for the improvement of interventions designed to promote sustained adherence to active lifestyle behaviors, facilitating its translation to community settings.

**Trial registration:**

April 20, 2023 – NCT05860621.

April 21, 2023 – https://doi.org/10.17605/OSF.IO/ZAQ9N

April 27, 2023 – UMIN000050945.

**Supplementary Information:**

The online version contains supplementary material available at 10.1186/s12885-023-11137-1.

## Background and rationale

Cancer is expected to become the leading cause of death and the single most important barrier to increasing life expectancy all over the world in the present century [1]. Still, the large increase in cancer survival rates, derived from advances in cancer detection and treatment, brought new challenges to cancer management and care [1]. Survivorship (i.e., from diagnosis to the end of life) is often related with long-term treatment side effects (e.g., fatigue), increased risk of cancer recurrence, and higher vulnerability to chronic diseases; all of which adversely affect cancer survivors’ quality of life (QoL) [2]. Hence, continuous care services need to be provided even long after the active treatment, placing increasing pressure on health care systems (USA costs estimated to be over US$450 billion in 2030) [3].

Breast cancer is the second most common cancer in Portugal and one of the three most common worldwide [4]. Breast cancer survivors diagnosed within the past five years were estimated to be 6.8 million worldwide and 155.5 thousand in Portugal [1]. About 75% of breast cancer diagnoses are hormone-receptor-positive [5], and often treated with aromatase inhibitors (AI), after active treatment, in post-menopausal women [6]. AI improve disease-free survival by 10–40% [7], but have several adverse side effects (e.g., arthralgia, osteoporosis, menopausal symptoms) that affect QoL [8]. Body image is often compromised in breast cancer survivors, negatively affecting daily functioning and QoL [9]. These side effects often lead to premature AI discontinuation, and eventually, to lower treatment efficacy and increased mortality [10].

Physical activity (PA) is safe and should be an integral and continuous part of care for all individuals diagnosed with cancer [11]. There is compelling evidence suggesting that PA plays an important role in improving longevity among cancer survivors [12]. PA effectively ameliorates short- and long-term adverse effects of cancer therapies (e.g., comorbid conditions), improves physical fitness, physical functioning and sleep, attenuates cancer-related fatigue, enhances body image and QoL, and decreases cancer recurrence and mortality [12, 13]. PA also allows women to benefit from endocrine therapy while being protected against the related risk of osteoporosis, fracture, and ultimately cancer recurrence or death [14]. Thus, PA in breast cancer survivors, and specifically among women on AIs, is paramount to improve health outcomes, QoL, and prevent therapy discontinuation.

However, the health system is overburden, and it does not have an integrated solution (that incorporates exercise programs) for cancer survivors who finish the active treatment and often feel overwhelmed by the necessity of self-managing treatment side effects on their own [15]. Thus, most cancer survivors fail to meet established guidelines for PA [16], not benefiting from exercise positive effects on health and QoL. Global (lack of motivation), practical (affordability) and health-related exercise barriers (fatigue, pain) are often reported at the individual level [15], but also barriers concerning the implementation of PA programs in oncological populations [17]. Additionally, the existence of community exercise programs and their integration into health care is still scarce [18, 19]. Still, healthcare professionals can have a strong impact on cancer survivors’ PA adoption [20]. Also, providing PA assessment, brief counseling, and referral as part of routine healthcare has been recommended in the WHO Global Action Plan for Physical Activity 2018–2030 [21].

Theory-based interventions, using evidence-based behavior change techniques (e.g., self-monitoring, goal setting or action planning) known to mediate long-term PA adherence [22], are effective in breast cancer survivors [23], but still scarce [24]. Also, most interventions fail to provide validated self-regulatory tools or explore meaningful links between PA and patients’ values and life aspirations to foster lasting behavior changes [25]. Prior research has shown that internal (better quality) forms of motivation play an important role in PA and behavior sustainability [26, 27], suggesting that self-determination theory (SDT) [28] can be a valid framework to promote sustained adherence to PA. We have also started establishing SDT relevance in the design and delivery of PA interventions [29, 30], confirming that a need-supportive intervention climate enhances people’s wellbeing and their ability to self-regulate and sustain behavior changes [31]. Brief counseling interventions to foster PA, involving an approach to motivations, barriers, preferences, readiness, and patient's opportunities to perform PA [32], have gathered evidence of effectiveness, compatible with clinically relevant increases in PA levels, in the general population [33]. However, there is still a lack of studies assessing the external validity of this type of interventions, when implemented in real-world settings, which limits the generalizability of such results [34].

In sum, although regular PA is a promising and safe way of helping cancer survivors navigate their disease, thus also alleviating the growing pressure on the health care system, most cancer survivors do not meet the recommended PA doses. It is a goal of this project to overcome the abovementioned shortcomings, by testing an intervention model informed by solid evidence and a robust theoretical rationale [28], provided by qualified exercise professionals (i.e., with a master in Exercise Sciences) – with the potential to add value to the treatment process, improving the therapeutic effect and safety of the exercise practice [35].

### Objectives

This paper describes the protocol of a pragmatic randomized controlled trial (RCT), PAC-WOMAN, targeting Portuguese post-menopausal women diagnosed with hormone-receptor-positive breast cancer, currently on Aromatase Inhibitors. This project primary aims are:a) To develop and test the long-term (16-month) effectiveness and cost-effectiveness of two 4-month group-based interventions, compared to a control waiting list, on promoting sustained changes in PA, sedentary behavior, QoL, and healthcare services’ use (primary outcomes)b) To compare the effects of both interventions (i.e., brief PA counseling vs. structured exercise) on long-term changes in our primary outcomes, as well as their cost-effectivenessc) Evaluate pretreatment moderators of 4-month and 16-month changes in PA, sedentary behavior, and QoLd) Identify critical motivational (theoretical) mechanisms of change in PA, sedentary behavior, and QoL, and explore their mediating role.

As secondary aims, we will study the impact of both interventions on AI therapy continuation, adverse events of treatment, disease-free survival, body composition, sleep quality, physical parameters (e.g., muscular strength), and psychosocial factors (e.g., body image). Furthermore, process evaluation will also be conducted seeking to analyze implementation facilitators and barriers.

### Methods: Participants, interventions, and outcomes

This protocol follows SPIRIT guidelines [36]. The SPIRIT checklist can be found in Additional File 1.

#### Rationale for the PAC-WOMAN trial

Supervised exercise programs may provide significant symptomatic benefit for patients living with or beyond cancer [11, 12], however their widespread implementation may be restricted by lack of facilities and funding. Indeed, there is a lack of effective PA interventions that are low-cost and can be implemented at scale and fully-embedded in a system [37]. Furthermore, once these exercise programs end, the sustainability of PA behavior and its subsequent health outcomes may be at risk. Thus, there is a need for effective, scalable, low-cost interventions to enhance the adoption and maintenance of regular PA. Brief counseling interventions that promote autonomous motivation for free-living PA, reinforcing self-regulation resources, may be an alternative, less expensive, solution. Evidence has suggested that brief counseling interventions may be as effective as more intensive interventions [38].

Indeed, there is an urgent need for affordable and practical interventions that can be delivered in real-world health care or community settings and have similar degrees of effectiveness [34]. A systematic review exploring differences between supervised exercise, home‐based exercise, and walking advice for intermittent claudication has not found clear differences in quality of life or self‐reported functional impairment between supervised exercise and free-living PA solutions, suggesting that the latter might also be important alternatives within PA promotion [39]. In line with this, brief PA counseling interventions, that are underpinned by theoretical models of behavior change and incorporate behavior change techniques, such as identifying barriers, self-monitoring, goal setting, and feedback provision, have been shown to increase PA in the general population [40, 41], as well in people with physical disabilities [42].

In sum, existing interventions targeting supervised exercise, although very effective in promoting short-term health outcomes [43], may be labor intensive and costly for staff and participants. Also, long term effects, once the intervention is finished, may be jeopardized given that supervised interventions may have the detrimental effect of patients’ internalization of the message that their condition is to be dealt with by procedures and techniques essentially under the responsibility and “steering” of an external expert (e.g., simply follow an exercise prescription), thus promoting an external locus of causality. In contrast, theory-based, evidence-informed brief counseling, delivered in a manner that reduces dependence on staff and facilitates self-regulatory skills, could alleviate time and financial barriers while promoting free-living, integrated into daily-life, PA/exercise. From a self-determination theory (SDT) perspective [28], lasting behavior change does not depend on complying with external demands for change but rather on accepting the regulation of change as one’s own. This requires internalizing the regulation of relevant behaviors and integrating them with one’s sense of self and one’s values and goals, so they can become the basis of autonomous regulation [27].

Thus, the PAC-WOMAN trial will test effectiveness and cost-effectiveness of these 2 types of interventions (supervised structured exercise vs. brief theory-based PA counseling) against a waiting-list, standard care, control group. This is of importance given that although an improved health profile (e.g., lower blood pressure, lower body mass index, improved tumoral biomarkers, cardiorespiratory fitness, physical function) is expected as a short-term result of the exercise intervention, the maintenance of the PA/exercise habits after the end of the intervention remains to be determined (and consequently its long-term health outcomes). On the other hand, will a brief motivational (SDT-based) intervention (lighter in time and resources) be enough to trigger a more physically active lifestyle? And will the potential behavioral, motivational, and self-regulatory changes endure in the long term? (With a sufficient magnitude to produce positive health outcomes?).

This contrast can be better understood considering the theoretical background of SDT [28], a macro theory of human motivation widely used to develop health behavior change interventions in multiple health domains and populations [26, 27]. This framework was selected because it accounts for the nature and function of motivation [28] and provides a theoretical explanation for both behavioral and psychological health issues in symptomatic populations [44]; issues that represent critical priorities in cancer survivor care [45]. SDT proposes that creating a need-supportive environment, that fulfills one’s psychological needs for competence, autonomy, and relatedness, will foster greater levels of autonomous (self-determined) motivations (i.e., self-endorsed reasons to perform a behavior, coherent with one’s core values and interests), which will in turn lead to long lasting behavior change [28]. Systematic reviews and meta-analyses provide empirical support for the use of SDT to develop PA behavior change interventions for adults in general [26, 27]. In cancer populations, a few correlational studies have provided support for SDT propositions, namely for the role of autonomy support and autonomous motivations on PA adoption and psychological wellbeing [46–48]. Still, SDT-based interventions targeting cancer survivors are only giving the first steps. To date, we could only find a pilot intervention study testing the positive effects of an autonomy-supportive exercise instructing style on breast cancer survivors’ wellbeing [49] and a very recent protocol for another pilot SDT-based intervention to promote PA via videoconferencing technology in young adults [50].

#### Study design and setting

PAC-WOMAN is a three-arm, superiority parallel-group, pragmatic randomized controlled trial (RCT), comprising a 4-month intervention period and a 12-month follow-up, that will be conducted to evaluate the effectiveness of two interventions aimed at promoting active lifestyles and improved quality of life in the long-term, albeit with different approaches (brief PA counseling vs. supervised structured exercise), compared to a control group.

Clinical trials can be conceptualized across a continuum from explanatory to pragmatic, with the former assessing if a program could work under ideal circumstances and the latter determining if it could work in real world conditions. Pragmatic trials are necessary for developing and testing interventions using the settings, resources, patients, and approaches to which they will ultimately be implemented [51, 52], thus ensuring that research funds have the greatest potential to impact patient and population health and have therefore been recommended [53].

This project will be conducted under real world circumstances at a main urban center served by several hospitals. Interventions were designed to take place at local gyms, with facilitated accessibility and appropriate equipment and facilities. Recruitment of participants is taking place via dissemination of the trial in main hospitals with oncology centers. A schematic description of the study phases and participant’s timeline, according to SPIRIT, is presented in Table 1. Participants will be enrolled in cohorts or “waves”. Ethics approval was obtained prior to study development/implementation from the faculty responsible for the study (FEFD: M25C21) and all recruitment hospitals (CAML: 285/21; HFF: 10/2022; IPOLFG: UIC/1499; CHLO: 2281). The trial is being conducted in accordance to the declaration of Helsinki for human studies from the World Medical Association [54].

**Table 1:**
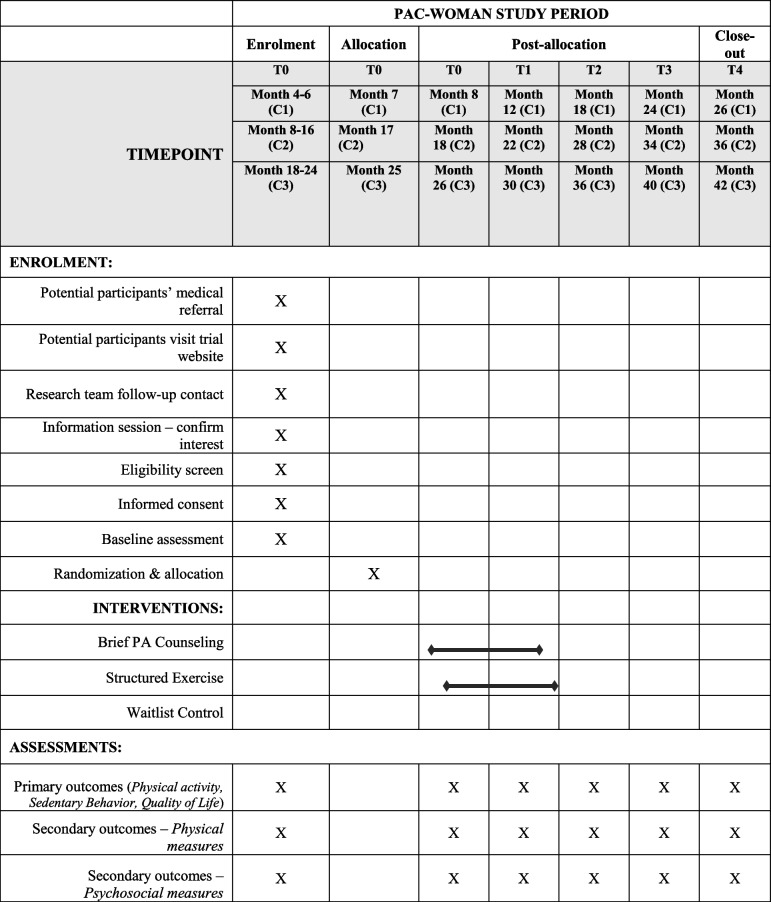
SPIRIT diagram flow for the PAC-WOMAN study

Legend: *C1* Cohort 1, *C2* Cohort 2, *C3* Cohort 3

#### Participants

Recruitment (ongoing) will aim for 122 Portuguese breast cancer survivors who have completed primary cancer treatment and are currently under aromatase inhibitors therapy and will subsequently be randomized to receive the brief PA counseling (PAC), the structured exercise program (StEx), or to a waiting list control group.

The inclusion criteria for entering this study are: 1) post-menopausal women, below 70 years old; 2) histologically confirmed hormone-receptor-positive breast cancer (stage I, II, III); 3) having initiated AI hormonal therapy following the primary treatment (surgery, radiotherapy, chemotherapy, etc.), at least 1 month before being enrolled; and 4) ECOG-Performance Status 0–1 (being able to perform light-to-moderate physical activities). The exclusion criteria are: 1) evidence of stage IV cancer or synchronous tumors; 2) uncontrolled hypertension, cardiac or pulmonary disease; 3) contraindications to exercise training according to the assistant doctor; 4) inability to provide informed consent; and 5) expected inability to fulfill the proposed program schedule.

Given the study design, sample size estimations were calculated for primary outcomes having one-way factorial analysis of variance with repeated measures as the reference statistical analysis. Based on previous research findings, and considering a 25% dropout, the sample size was estimated in 98 participants to detect a small to moderate effect size (α = 0.05; statistical power = 0.80) using G*Power 3.1. Consensual criteria on sample size needs for mediator/moderator analyses are not consensual and depend on the procedure and number of indicators and pathways in the models; therefore, we increased the estimated number of participants in 25%, obtaining a final estimated sample size of 122 participants.

#### Recruitment and screening

Ongoing recruitment is taking place via study dissemination and referral from the medical teams of several main hospitals (Hospital de Santa Maria, Hospital Professor Doutor Fernando Fonseca, Instituto Português de Oncologia – Lisboa, Hospital de São Francisco Xavier, and CUF Tejo/Descobertas). A website (https://pac-woman.ulusofona.pt/) and a Facebook page (https://www.facebook.com/projeto.pacwoman) were created to disseminate the project and help with the recruitment and dissemination process. Informative brochures for physicians and patients were produced and delivered in recruiting hospitals.

The post-COVID-19 pandemic period of this study is expected to create additional difficulties to recruitment [55–58]. As a result, additional, more direct, strategies like contacting patient associations, circulating Facebook adds tailored to women in the study age range in special cancer group pages, requesting public figures that survived breast cancer to disseminate our study, distributing flyers directly to the target population in cancer-related sport events, or advertising our study in TV/Radio shows, are also being considered.

Patients referred by the medical teams are, then, contacted by phone by the research team to receive detailed information about the study. Alternatively, in case of preference, patients can also call the research team directly, or manifest interest via email or Facebook. In all cases, after confirming eligibility criteria and interest to participate, patients are asked to assist to an informational session and, in case of interest, sign the inform consent form. Participants are informed that they are under no obligation to participate and may withdraw their consent at any time. Additional consent provisions for collection or sharing of participant data are also obtained. A manual on recruitment and screening procedures was produced.

#### Randomization and blinding

After signing the informed consent and completing the baseline assessments, participants are randomly allocated to one of the three arms. Allocation is being made according to an automated computer-generated randomization scheme performed by a member of the research team not directly involved with participants. Adjustments in the allocation ratios might be made if recruitment takes longer than expected and interventions are required to start for timely reasons. Group assignment is only communicated to participants after all baseline testing is finished.

Participants and research team staff are unaware of group allocation at the time of recruitment and baseline assessments because randomization is only performed after participants have completed baseline assessments. This study will involve behavior change interventions, preventing any concealment of group allocation after randomization from either participants or intervention counsellors. Nonetheless, participants will be blinded to the study rationale. Also, to minimize bias, physical activity and sedentary behavior (primary outcomes) are objectively assessed using accelerometers and psychometric data are collected by self-report and introduced in the Qualtrics platform in its raw state. All outcome data will be kept blinded until the final data entry for the entire study is completed.

#### Interventions

The brief PA counseling (PAC) program will comprise 8 sessions (120 min each), every fifteen days, addressing the following themes: reasons to change, an introduction to the PAC-WOMAN program and principles, types of physical activity and their benefits, strategies on how to become more active and less sedentary, how to safely practice exercise at home, the importance of social support for doing more physical activity, barriers and facilitators for becoming more active, development of coping plans and strategies to overcome those barriers, establishing SMART goals, self-monitoring, medical aspects related to symptom management in breast cancer survivors, body image and self-acceptance, sharing experiences with role models, and re-evaluating action plans.

To increase the potential for long-term behavior change, it is important to target theoretically proposed mechanisms of change and incorporate evidence-based behavior change strategies. Self-Determination Theory (see Rationale for the PAC-WOMAN trial), and several evidence-based behavior change techniques, will be used throughout the intervention. The goal will be to empower participants to develop self-regulation resources, fueled by autonomous motivation to integrate physical activity into daily lives, even in the face of adversity, and thus sustain changes in physical lifestyle. The basic psychological needs of autonomy, competence, and relatedness [28] will be supported in all sessions and intervention materials. More specific SDT-based behavior change strategies will include the provision of choice, meaningful rationale, connection between participants’ health behavior and deep inner values, goal setting, action planning, self-monitoring, coping planning, weighing pros and cons of change in the context of held values and life priorities. Group-based sessions will allow the exchange of experiences, feelings, difficulties, and effective solutions to overcome them, in a respectful, non-judgmental, and supportive environment. The portfolio of strategies, and intervention motivational climate, is in accordance with systematic reviews in this regard [41]. Exercise professionals, previously trained in brief counseling skills for health behavior change and its implementation, will deliver the sessions.

A participants’ manual was developed, including a toolbox supporting each session. An activity tracker will be provided to all participants to facilitate self-monitoring. An Exercise Booklet was also created, including different exercises that can be performed at home, accompanied by QR codes with complementary instructive videos. An invested role model (a women previously diagnosed with breast cancer) will illustrate the exercises in the booklet during one of the sessions to show the easiness of their completion. Participants will be prompt to use the booklet as they see fit, during their daily lives, in an integrated fashion (passible to be integrated in the long term) and at a self-selected pace.

The structured exercise program was designed based on the most recent guidelines for exercise prescription and safe practice in cancer populations [12, 19, 59]. A group-based supervised program comprising 32 sessions (over 4 months), lasting 90 min each, and taking place twice a week, was developed. The program combines mobility, aerobic, and strength exercises, respecting a periodization model of four mesocycles of 8 sessions, with progressive intensity and complexity, following the periodization model for each exercise component. Once every 15 days, a thematic group class will be offered to participants, so that they can explore different physical activities and enhance their exercise experience.

Each session will be organized into four components: (i) warm-up—a 10 to 30-min combination of articular mobility exercises and body weight activities; (ii) muscular strength—30 to 45 min, 6–7 large muscle group exercises with an intensity of 60–80% of one maximal repetition (10–15 repetitions, 2–3 sets); (iii) aerobic training—20 to 30 min of a cardiorespiratory workout modality chosen according to participant’s preferences (treadmill, elliptic, bike and row machine) with an intensity of 40–60% of reserve heart rate; (iv) cool-down (5 min). Group sessions will be provided in a person-centered, positive interpersonal environment, by qualified exercise professionals.

An individualized target zone intensity will be previously determined for each participant, based on a preliminary physical evaluation, and monitored during exercise sessions using perceived exertion scales [60] and heart rate monitors (*Polar Club software*). During the sessions, participants’ potential exercise-related adverse effects will be monitored and registered, as well as individual attendance and intensity zone compliance. If participants present any limitations during the session, the exercise prescription will be adapted to respect their individual needs.

Patients allocated to the control group (i.e., waiting list) are asked to continue their daily routines, receiving their standard medical care. No additional instructions will be given to the control group. At the end of the study, the control group will be offered the structured exercise program for 4 months. This type of control group was chosen to allow us to clearly establish whether the tested interventions are effective and superior to no intervention at all.

#### Intervention facilitators training

PAC intervention facilitators will receive training on motivational counseling skills, congruent with a person-centered, SDT-based approach, by a certified trainer. A detailed intervention manual, to support the delivery of the sessions, was produced. It details all the components of each session, including: a) goals and components for each session; b) the importance of each component (the “why”); 2) tips for implementation (the “how”); 3) time allocated to each component/task; 4) materials needed. Intervention counsellors will complete initial training sessions, performing mock counseling encounters with participants concerning the sessions to be delivered. Furthermore, ongoing supervision will also be provided, via weekly meetings to discuss session implementation challenges, deviations or required adjustments to the manual (planned activities for each session), feedbacks, and challenges from participants’ processes.

The supervised structured exercise program was implemented by certified exercise professionals, with a master’s degree in Exercise and Health, and a recognized Advanced Qualification in Physical Exercise and Cancer (provided by Liga Portuguesa Contra o Cancro). An exercise intervention manual was created to guide the interventionists in the implementation of the program. This manual includes information about 1) safety measures and alert signs and symptoms the interventionists should be aware of, 2) the structure of the mesocycles in terms of exercise doses (volumes, intensities), exercises, and specific goals, 3) planned thematic group classes, 4) required equipment and clothes.

#### Criteria for discontinuing or modifying allocated interventions

This is an adaptive, pragmatic trial, subject to real world conditions, thus adaptations may be needed. Data retrieved during trial implementation (mainly from process evaluations, via focus groups with participants at the end of each cohort and interviews with session facilitators, staff from recruiting hospitals and stakeholders) will help to decide the need to adjust aspects of the study as it continues (without undermining the validity and integrity of the trial) [61]. For instance, adjustments might be made in the order or interval between the sessions, based on participants' feedbacks; group allocation ratios might be adapted if recruitment takes longer than expected and, for timely purposes.

It is important to highlight that the PAC-WOMAN trial comprises two non-invasive interventions, with minimal risk of harm. Participants will go through a comprehensive assessment at baseline and those enrolling in the structured exercise program will be provided with a tailored, individualized, prescription, with a gradual progression, always adapted to their needs and capacities. Participants will be asked to report any adverse event and we will keep a close contact with the medical team, always assuring participants’ safety. Also, a personal accident insurance will be contracted and triggered whenever needed.

#### Strategies to improve adherence to interventions and follow-up assessments

One of the major risks in this study is the low compliance of patients to the interventions and assessments. Although most cancer survivors manifest interest in participating in physical activity [15], their lifestyle and implemented routines may interfere with their participation. In addition, a severe decline in physical function is expected to occur during AI therapy, which could potentially lead to increased dropout rates. Nonetheless, the research team includes medical oncologists, physiatrists, and qualified exercise professionals that will assure a close proximity with participants throughout the course of the intervention, assess their experiences and opinions about the program and ways of improving it, and provide a rationale for all the activities involved in the intervention, making sure they continue committed to the trial’s protocol. Moreover, a qualitative analysis (through focus groups) will be applied to enhance the understanding of the patients’ perspectives about: the impacts of the intervention in their daily life; benefits and disadvantages of participation; functional aspects (e.g., dose, procedures, structure) of the intervention; and suggestions of improvement. To increase adherence to follow-up assessments, a session on healthy nutrition for cancer survivors will be offered at this point, as this is a topic that gets a lot of interest among women. Also, at each assessment timepoint, a treat will be offered to participants to stimulate their adherence to the evaluations (e.g., using the gym pool for the day of assessments; a week voucher to try out different gyms), scheduled at a time of their convenience. Participants will also receive an early reminder of the approaching assessment point, and then gentle reminders confirming the date, time, and location of their upcoming visit on the day before.

#### Assessments

Participants will be assessed at four time points: baseline (T_0_), 4 months (intervention’s end; T_1_), and 10 and 16 months (T_2-_T_3_; during follow-up). A schematic description of all the measurements by timepoint is presented in Table 2. Assessments will take place in standardized conditions, at the exercise setting, in a calm comfortable environment, in small groups, with a group of study technicians attending every assessment period. At each assessment point, the research staff provides participants with an accelerometer, which they are required to wear for a week, and schedules an evaluation visit to the exercise setting, where participants complete the full battery of assessments, described below, and according to the assessment plan for that timepoint. An individual report with the assessment results will be provided to each participant at the end of each assessment period is complete. Participants will be contacted close to each assessment point to determine any changes in their contact or personal information that may have occurred, especially in the fulfillment of the eligibility criteria.

**Table 2:** Measurements’ timeline

	**Intervention**		**Follow-up**	
	**T0 Baseline**	**T1 4 months**	**T2 10 months**	**T3 16 months**
**Primary outcomes**				
Physical activity/sedentary behavior (objective and self-reported)	X	X	X	X
Lifestyle physically active choices	X	X	X	X
Quality of Life	X	X	X	X
Healthcare use	-	X ^a^	-	-
**Secondary outcomes – Physical Measures**				
Body composition	X	X	X	X
Cardiorespiratory fitness	X	X	X	X
Muscular strength	X	X	X	X
Upper limbs flexibility	X	X	X	X
Physical function	X	X	X	X
Adverse events	X	X	X	X
**Secondary outcomes—Psychosocial Measures**				
Perceived intervention climate	-	X	-	-
Exercise motivational regulations	X	X	X	X
Exercise needs satisfaction/frustration in exercise	X	X	X	X
Affective response to exercise	X	X	X	X
Exercise self-efficacy	X	X	X	X
Self-regulation skills	**-**	X	X	X
Subjective Pain	X	X	X	X
Sleep quality	X	X	X	X
Body image	X	X	X	X
Depressive symptoms	X	X	X	X
Psychological well-being	X	X	X	X
**Potential covariates**				
Treatment-related factors and demographics	X	-	-	-
AI Therapy length/continuation	X	X	X	X
Food intake	X	X	-	X
**Process evaluation (intervention groups-only)**				
Participants focus groups	-	X	-	X

^a^Assessment performed 6 months after intervention’s start

##### Primary outcomes

Objectively measured physical activity. Objective measures of PA are obtained through Actigraph GT9X accelerometers. The accelerometer is worn on the wrist and participants are instructed to wear the devices continuously for 7 days (day and night) during their daily activities and sleep. The sampling unit (epochs) is set to 1 s to facilitate data analysis and ensure sufficient sensitivity for low intensity activities. The participants accelerometer counts are categorized into activity levels (sedentary, light, moderate and vigorous) according to the cut-off points established by Montoye et al. [62].

Self-reported physical activity. The Short-Form of the International Physical Activity Questionnaire (IPAQ) is used to assess self-reported PA and sedentary behavior. The 9-item IPAQ measures the weekly frequency and duration of PA across three specific intensities (i.e., light, moderate, and vigorous), and time spent sitting during week and weekend days. Scores for total PA and discriminated by intensity, and for total sitting time, will be derived from the data collected (min/week) [63]. The Activity Choice Index (ACI) is employed to assess physically active lifestyle behaviors. The frequency of self-reported activities, which represent active choices made in daily routines, over the last month, are assessed using a 6-item (e.g., “using stairs instead of escalators or lifts”; “walking instead of driving or taking public transport”; “choosing to do things by hand instead of using mechanical/automatic tools”). The items are scored according to a 5-point scale, from one ("Never") to five ("Always") [64].

Quality of life. The European Organization for Research and Treatment of Cancer Quality of Life Questionnaire Core 30 (EORTC QLQ-C30) and its breast cancer module (EORTC QLQ-BR23) are used to measure changes in participants’ quality of life throughout the intervention and follow-up phases, and have previously demonstrated adequate reliability [65, 66]. EORTC QLQ-C30 comprises 30 items, organized in 8 multi-item functional (physical, role, emotional, cognitive, and social) and symptom (fatigue, pain, and nausea) subscales, one global health status and quality of life (QOL) subscale, and 6 single items (dyspnea, insomnia, appetite loss, constipation, diarrhea, and financial difficulties). Breast cancer-related symptoms and side effects are assessed with EORTC QLQ-BR23 module, comprising 23 items, organized in 5 subscales (i.e., body image, sexual functioning, systemic therapy side effects, breast symptoms, and arm symptoms). Five items specifically related to joint, bone, and muscle pain/discomfort, derived from the new EORTC QLQ-BR45 [67], were added provided the specificity of aromatase inhibitors’ side effects.

Healthcare use. Data is collected on healthcare resources use during the study period, namely number and type of consultations, drugs, medical tests and exams, in-patient stays, and day care sessions. Absenteeism is assessed using participants’ reports of their number of absence days or percentage of normal working hours worked, valued at patients’ hourly wage. These resources will then be valued using usual official sources.

##### Secondary outcomes: Physical measures

Body composition. Bioelectrical Impedance (Impedimed, Australia) is used under standardized conditions, by experienced technicians and overseen by the research team. Body weight is measured with a digital scale (SECA, Germany). Height is measured with a balance-mounted stadiometer. Body mass index in kilograms per square meter is calculated from weight (kg) and height (m). Waist circumference is measured according to the NIH protocol [68].

Upper limb perimeters. Right and left limb perimeters are measured in five anatomical points along the arms – hand knuckles with closed fingers, wrist, forearm (10 cm below the elbow line), elbow and arm (10 cm above the elbow line). Lymphedema risk is assessed through the difference between the right and left limb (hand, forearm, and arm) perimeters. A difference of 3 cm between limbs in one of the perimeters indicates risk of developing lymphedema.

Cardiorespiratory fitness. A submaximal, 8-min, single-stage walking test on a treadmill is performed to measure cardiorespiratory fitness. The test involves a 4-min warming up at a self-selected speed, at 50–70% of the individual's age-predicted maximum heart rate, and 4 additional minutes at a 5%-increased workload [69]. The steady-state heart rate at this workload and the treadmill speed, together with participants’ age and gender, are used to estimate VO2max.

Muscular strength. Handgrip and maximal strength tests are performed to assess muscular strength. Participants are instructed to hold the handgrip with their maximal strength. Maximal muscular strength is determined for chest press, horizontal seated row and leg press, using a 10-repetition maximum (10 RM) test [70]. After a standardized warm-up, gradual load increases are made until the maximum weight lifted through a full range of motion is recorded as 10 RM.

Upper limbs flexibility. Shoulder mobility protocols is used to measure flexibility. Angular measures [71] of shoulder flexion and abduction are taken on both sides using a goniometer. Linear measures are taken using the Back Scratch protocol [72] on both shoulders with a SECA measuring tape.

Physical Function. The Sit to Stand, Timed Up and Go and Stand on one-foot tests are employed to assess physical function. The Sit to Stand Test consists of standing and seating in a chair as many times as possible with arms crossed over the chest in 30 s [73]. The Time Up and Go Test is used to assess mobility by measuring the time a person takes to rise from a chair, walk 2.44 m, turn around, walk back to the chair, and sit down [74]. For the Stand on one foot test [75], participants are instructed to stand on one foot with their eyes open (both sides are tested) for a maximum of 20 s and have their time recorded.

Adverse events of anticancer treatment. A simplified version of the Common Terminology Criteria for Adverse Events (CTCAE v.5), using its broader categories (i.e., cardiac disorders, respiratory disorders, nervous system disorders, infections, skin tissue disorders, musculoskeletal and connective tissue disorders, and immune system disorders) is used at each timepoint to assess the occurrence of any adverse events in the previous 3 months.

##### Secondary outcomes: Psychosocial Measures

Perceived intervention climate. Participants’ perception of the facilitators interpersonal behaviors is measured with the Interpersonal Behaviors Questionnaire (IBQ) [76], a 24-item instrument including three support subscales—perceived autonomy, competence, and relatedness support – and three thwarting subscales – perceived autonomy, competence, and relatedness thwarting. Responses are given on a 7-point Likert scale, ranging from 1 (“do not agree at all”) to 7 (“completely agree”).

Exercise motivational regulations. The 24-item Behavioral Regulation in Exercise Questionnaire-3 (BREQ-3) [77] will be used to measure the six forms of motivation proposed by self-determination theory – amotivation, external regulation, introjected regulation, identified regulation, integrated regulation, and intrinsic motivation. Responses are given on a 5-point Likert scale, ranging from 1 (“Strongly Disagree) to 4 (“Strongly Agree”).

Exercise needs satisfaction/frustration. The 24-item Basic Psychological Need Satisfaction and Frustration Scale (BPNSFS) [78] will be used to assess satisfaction/frustration of the three basic psychological needs (autonomy, competence, and relatedness) for exercise. Responses are given on a 5-point Likert scale, from one (“Totally disagree”) to five (“Totally agree”).

Affective response to exercise. The Feeling Scale (FS) [79, 80], is an 11-point scale ranging from -5 (“Very bad”) to + 5 (“Very good”), which assesses the affective valence of exercise.

Exercise self-efficacy. The 9-item Modified Bandura’s Exercise Self-Efficacy Scale will be used to measure how certain participants are/were that they would practice exercise under different conditions or restrictions [81]. Responses are given on a 4-point Likert scale, from 1 (“Very sure”) to 4 (“Not at all sure”).

Self-regulation skills. Action planning (i.e., when, where, what to do, and how often exercise) and coping planning (i.e., how to cope with setbacks and what to do to act according to one’s intentions to exercise) will be assessed the Action Planning and the Coping Planning scales, comprising 5 items each [82]. Action control will be measured with 6 items addressing its different facets, (i.e., self-monitoring, awareness of standards, and self-regulatory effort) [83]. All items are answered on a 4-point Likert scale from 1 (“Completely disagree”) to 4 (“Totally agree”).

Subjective pain. Single items of the Brief Pain Inventory (BPI) will be used to assess pain severity “on average” and “right now”. The BPI is scored on a 10-point scale from 0 (“No pain”) to 10 (“Pain as bad as you can imagine”) [84]. The 7-item Pain Disability Index (PDI) will be used to evaluate the impact and interference of pain on participants’ daily activities and functioning (i.e., family and home responsibilities, recreation, social activity, occupation, sexual behavior, self-care, and life-support activities). Each item is rated on a 10-point scale, from 0 (“No Disability”) to 10 (“Worst Disability”) [85, 86].

Sleep quality. The 19-item Pittsburgh Sleep Quality Index [87] will be used to measure sleep duration and sleep disturbance components of sleep quality. Each item is rated on a 4-point scale, ranging from 0 (“Not during the past month”) to 3 (“Three or more times a week”).

Body image. The 10-item Body Image Scale (BIS) [88] will be used to assess participants’ affective (e.g., feeling self-conscious), behavioral (e.g., difficulty at looking at the naked body), and cognitive (e.g., satisfaction with appearance) dimensions of body image. Responses are given on a 4-point Likert scale, from 0 (“Not at all”) to 3 (“Very much”).

Depressive symptoms. The 7-item depression subscale from the Hospital Anxiety and Depression Scale (HADS) [89, 90] will be used to measure depression. Responses are given on a 4-point Likert scale, from 0 (“Never”) to 3 (“Most of the time”). HADS scores can be categorized into normal (0–7 points), mild depression (8–10 points), moderate depression (11–14 points) and severe depression (15–21 points).

Psychological well-being. Four items, asking participants to rate their overall satisfaction with life, optimism, and purpose of life and daily activities, will be used to measure the various dimensions of psychological well-being [91]. Responses are given on a 10-point scale, from 0 (“Not at all”) to 10 (“Completely”).

##### Potential covariates

Demographics such as age, education level, professional status, marital status, smoking history, alcohol consumption, medical factors (i.e., breast cancer stage, cancer treatment types, time since diagnosis, time since treatment, length of AI therapy, comorbid conditions, and current medications, blood pressure, and resting heart rate) will be considered as potential covariates. Participant’s medical history will be confirmed by personal medical reports brought by participants. Given that dietary habits and caloric intake, unrelated to our interventions, may affect body composition, participants will complete the adapted version of the Dietary Instrument for Nutrition Education (DINE) [92]. DINE measures self-reported dietary behavior by examining the frequency of intake of 19 food/drink items (e.g., cheese, burgers/sausages, beef, pork/lamb, chips, bacon/ham, savory pies/snacks, fruits, vegetables, sweets, biscuits, sugary drinks, milk). Alcohol consumption and frequency of breakfast will also be assessed.

#### Process Evaluation

A process evaluation will be embedded in the RCT to provide insight into whether the two intervention programs are being delivered as intended; identify potential barriers and facilitators of implementation; explore why they did or did not produce the intended outcomes; and assess participants’ and interventionists’ experiences of the program. At post-program and final follow-up measurements, the two intervention groups will be asked to join focus groups focusing on: experiences with the program, toolkit, and resources available; which elements they found most useful; the extent to which they are still interacting with their peers after the program has ended; and the perceived impact of the programs on their lives (barriers and facilitators of behavior change). A logbook will also serve to assess easiness of implementation and adherence to sessions. This logbook, fulfilled weekly by the session facilitators, includes session attendance, implementation of each session components (vs deviations), and additional comments/unexpected events. Interviews with session facilitators and healthcare providers involved in recruitment, exploring their experiences with the program (barriers and facilitators) will also be conducted at intervention´s end.

#### Statistical methods

Factorial Anovas with repeated measures, adjusting for potential covariates (e.g., primary treatment, length of AI therapy), will be conducted for primary and secondary outcomes. The analysis will be intent-to-treat to include compliance effects in the overall assessment. Last Observation Carried Forward will be used to impute missing values. A per-protocol analysis will also be conducted without participants who do not attend at least 80% of the sessions. Normality plots and Kolmogorov–Smirnov tests will be used to test normality of outcome variables. If substantial departure from normality is found, square root log transformations will be computed. If normality is still not satisfied, non-parametric tests (e.g., Kruskal–Wallis) will be employed. As the experimental design is factorial, multiple comparison adjustments will not be appropriate.

Mediators of change, i.e., mechanisms by which participants change their lifestyle behaviors and QoL (vs. controls), will be explored using structural equation modeling (AMOS 18.0) and multiple mediation analysis (PROCESS macro v. 3.3 for SPSS). Putative candidates will include motivational (e.g., autonomous motivations) and self‐regulatory (e.g., action planning) variables. Mediation is said to occur when the causal effect of an independent variable (X) on a dependent variable (Y) is partially or fully explained by a mediator (M) [93]. Indirect effects will be tested using Preacher and Hayes’ procedures [94]. Bootstrapping with 5000 samples and 95% CI estimates for indirect effects will be calculated and assumed significant (α = 0.05) if the CI does not encompass zero [95, 96]. The ratio of the indirect effects to the total effects (P_*M*_) will be calculated to express the strength of the mediation effects [95]. Moderators, i.e., baseline characteristics that have an interactive effect with the intervention on the outcome(s), will be assessed to better understand individual differences in intervention effects [97, 98]. Moderation effects on primary outcomes will be conducted at intervention or follow-up’s end.

A cost-effectiveness analysis will be conducted, from the NHS and societal perspectives, in comparison with the “no intervention” arm. The time horizon of the study will coincide with that of the intervention follow-up, so that no specific modeling tools will be used. Quality-adjusted life years (QALYs) will be estimated using EORTC QLQ-C30 collected during the intervention, mapped to EQ-5D using mapping algorithms from the literature [97]. Intervention costs will be estimated using a bottom-up approach [99]. Attendance to PA counseling and exercise sessions will be registered; exercise physiologists’ time-related costs will be estimated using their gross hourly salaries, and equipment and disposables’ costs through invoices. Healthcare resources will include consultations, drugs, tests and exams, in-patient stays and day care sessions, valued using usual official tariffs. Absenteeism will be assessed using participants’ reports of their number of absence days or percentage of normal working hours worked, valued at patients’ hourly wage. The incremental cost-effectiveness ratio (ICER) will be calculated as the difference in costs divided by the difference in QALYs between intervention and control groups.

#### Data management

All collected data will be kept strictly confidential. The data obtained within the trial will be computerized and encrypted in a database, not containing any identifying elements of the participants. At the clarification session, after manifesting intention to participate and providing written informed consent, a unique ID code will be attributed to each participant. From that moment on, all data inserted in the databases will not be directly linked to the participant’s name, not allowing their personal identification. A dataset will be created for each timepoint. These datasets will be maintained by those responsible for the investigation on a secure server of CIDEFES-UL, for 10 years, and used for research purposes only. Datasets for specific analysis/sub-studies will contain only the necessary study variables plus demographic indicators and will be provided to the research team members upon request to the principal investigator.

#### Oversight and monitoring

##### Adverse event reporting and harms

All subjects will be followed up for adverse event collection, related and non-related to the intervention (when applicable), through self-reporting at the beginning of each session, and at each assessment point visit using a questionnaire specifically built for this study, based on the Common Terminology Criteria for Adverse Events (CTCAE v.5). In addition, between sessions or assessment visits, participants will be asked to contact the clinical team in case of difficulties.

The research team will meet regularly once a week to discuss participants and intervention progress, emerging challenges and required adjustments to the sessions. These meetings will also serve to monitor data on outcomes and adverse events, and to oversee participants’ safety. A data monitoring committee will not be needed for the PAC-WOMAN trial, given that both interventions are non-invasive with minimal risk of harm. In addition, participants will be protected by a personal accident insurance, activated whenever needed.

##### Plans for communicating important protocol amendments to relevant parties (e.g., trial participants, ethical committees)

Amendments to the protocol will be promptly communicated to participants when related to their direct involvement in the study or to data manipulation. If necessary, the written informed consent form will be amended. The principal investigator will notify the collaborating institutions (e.g., hospitals, gym) of any relevant changes to the protocol, as well as the funding agency. Amendments to the protocol will be submitted for approval by the Ethic committees of the involved institutions, if justified. Updates to the published protocol will be made, if necessary, and dated.

### Dissemination plans

The trial results will be published in international oncology, behavioral medicine, and physical activity scientific journals and presented at national and international conferences.

Besides dissemination of results, the scaling up of the interventions (if proven successful and cost-effective) will also be pursued. Indeed pragmatic trials, such as PAC-WOMAN, tend to produce results that have high applicability (external validity) for participants and decision makers [100]. This will be key for the potential future widespread implementation via relevant stakeholders. CRediT authorship criteria will be followed.

## Discussion

This project is set in the context of a remarkable increase in cancer survival rates, which are associated with long-term adverse side effects, increased risk of cancer recurrence, higher susceptibility to chronic diseases, and poorer quality of life [2]. Specifically, hormonal therapy with aromatase inhibitors is frequently used to treat hormone-receptor-positive breast cancer (75% of all cases), but it has multiple unfavorable effects, resulting in premature therapy discontinuation/switch. Continuous care is thus required even long after the primary treatment, placing a huge economic burden on the health care system [3].

Physical activity is safe and can help breast cancer survivors navigate their disease, attenuating the negative effects of AI therapy and improving quality of life. However, most cancer survivors fail to perform/sustain adequate PA levels, especially in the long-term, possibly due to the absence of appropriate exercise programs, led by qualified exercise professionals, and integrated in the health care services or available in the community. Hence, cancer survivors who finish primary treatment, feel overwhelmed by the necessity of self-managing treatment side effects on their own [15].

Theory-based interventions, using evidence-based behavior change techniques, aimed at promoting long-term health behavior change in breast cancer survivors are effective, but remain scarce, predominantly focused on short-term adherence/outcomes, and are resource demanding. Prior research has shown that autonomous (self-determined) motivations play an important role in long-term, sustained, PA adoption [26, 29, 30], supporting the use of Self-Determination Theory [28] as a valid framework. A person-centered, need-supportive intervention climate enhances individuals’ wellbeing, body image, and their ability to self-regulate and sustain behavior changes [31, 101, 102]. In cancer populations, a few correlational studies have provided support for SDT propositions, namely for the role of autonomy support and autonomous motivations on PA adoption and psychological wellbeing [46–48], though SDT-based interventions are just starting to emerge. In a recent systematic review of theory-based interventions targeting dietary and/or PA changes in cancer survivors, we could not find a single intervention based on Self-Determination Theory in this population (Rodrigues et al., in press). In effect, to date, we could only find a pilot intervention study testing and confirming the positive effects of an autonomy-supportive exercise instructing style on breast cancer survivors’ wellbeing [49] and a recent protocol of another pilot SDT-based intervention designed to promote PA via videoconferencing technology in young adults [50]. Also, skills such as self-monitoring, goal setting or action planning have been identified as mediators of long-term PA [22] and as core features of effective behavior change interventions in breast cancer survivors [23], but most interventions fail to promote the acquisition of proven effective self-regulatory skills. Concurrently, and although supervised exercise programs may provide significant benefits for patients from several chronic conditions, including cancer [11, 12], their widespread implementation is resource demanding, may be restricted by lack of facilities and funding, and may not be sufficient to create PA/exercise habits in the long run. Indeed, there is a lack of effective PA interventions that are low-cost and can be scale-up and fully embedded in the health care system.

To the best of our knowledge, PAC-WOMAN is the first study using Self-Determination Theory as a way of promoting the sustained adoption of an active lifestyle among cancer survivors, by offering a person-centered approach that fosters individuals’ sense of autonomy, competence and relatedness and explores meaningful links between PA and patients’ values and life aspirations, at the same time it stimulates the acquisition of validated self-regulatory skills (e.g., self-monitoring, goal setting or action planning). The significance of the PAC-WOMAN study is further supported by its focus on long-term adherence, along with the investigation of motivational mechanisms mediating changes in PA, sedentary behavior, and quality of life and of putative moderators explaining to whom these two interventions might work best. Finally, PAC-WOMAN is the first RCT study testing the long-term effectiveness and cost-effectiveness of a more traditional exercise program vs. a brief theory-based PA motivational counseling (against a control group). This is of importance given that although a larger improvement in health/fitness outcomes is expected as a short-term result of the exercise intervention, the maintenance of PA/exercise habits in the long term remains to be determined. On the other hand, will a brief motivational (SDT-based) intervention, lighter in time and resources, be enough to induce behavioral, motivational, and self-regulatory changes, and trigger a more physically active lifestyle?

This study is expected to have an immediate benefit to enrolled breast cancer survivors, and a direct impact on the improvement of interventions designed to promote adherence to active lifestyle behaviors. Dissemination of project-related interventions into the community, using community resources in close collaboration with the health care service, will provide much-needed cancer survival management tools that empower patients to maintain health-behavior changes for life, in a familiar social (non-medical) environment. If successful, this study will contribute to a decrease in NHS healthcare use, liberating resources for other needs.

### Trial status

Recruitment began in January 2022. Due to the COVID-19 pandemic, recruitment rates were very low. On April 27, 2023, 78 women had been screened by our research team, 68 had accepted to participate and had been randomized. Recruitment is still ongoing and the approximate date to be completed is December 2023. The trial final follow-up is expected to occur in February 2025. The trial is expected to be completed in August 2025.

## Supplementary Information

**Additional file 1.**

## Data Availability

Anonymized trial data will be available from the corresponding author upon reasonable request for non-commercial research purposes.

## Abbreviations

AI: Aromatase inhibitors
PA: Physical activity
QoL: Quality of life
SDT: Self-Determination Theory
RCT: Randomized controlled trial
FEFD: Faculdade de Educação Física e Desporto
CAML: Centro Académico de Medicina de Lisboa
HFF: Hospital Fernando da Fonseca
IPOLFG: Instituto Português de Oncologia—Lisboa
CHLO: Centro Hospitalar Lisboa Ocidental
CUF: Unidades Hospitalares CUF
PAC: Brief physical activity counseling
StEx: Structured exercise program
IPAQ: International Physical Activity Questionnaire
ACI: Activity Choice Index
EORTC QLQ-C30: European Organization for Research and Treatment of Cancer Quality of Life Questionnaire Core 30
EORTC QLQ-BR23: European Organization for Research and Treatment of Cancer Quality of Life Questionnaire Breast Cancer Module
EORTC QLQ-BR45: European Organization for Research and Treatment of Cancer Quality of Life Questionnaire Breast Cancer Module
NIH: National Institute of Health
RM: Repetition maximum
CTCAE v.5: Common Terminology Criteria for Adverse Events
IBQ: Interpersonal Behavior Questionnaire
BREQ-3: Behavioral Regulation for Exercise Questionnaire
BPNSFS: Basic Psychological Need Satisfaction and Frustration Scale
FS: Feeling Scale
BPI: Brief Pain Inventory
PDI: Pain Disability Index
BIS: Body Image Scale
HADS: Hospital Anxiety and Depression Scale
DINE: Dietary Instrument for Nutrition Education
QALYs: Quality-adjusted life years
ICER: Incremental cost-effectiveness ratio
CIDEFES: Centro de Investigação em Desporto, Educação Física, Exercício e Saúde
